# Diphtheria, Bacteria, and Dr. Wells

**Published:** 1881-12

**Authors:** Rollin R. Gregg

**Affiliations:** Buffalo, N. Y.


### DIPHTHERIA, BACTERIA AND DR. WELLS.

ROLLIN R. GREGG, M. D., BUFFALO, N. Y.

*Editor of the* HOMŒOPATHIC PHYSICIAN :

The November number of your Journal has just come to hand, and I have read Dr. Wells' ridiculing and characteristic reply to my criticism of his very unjust and wholly unprovoked attack upon me, in your June number.

Dr. Wells' evident intent to misrepresent me has led to a question of veracity between us. He said in his June criticism that I had given no proof of what I claimed, and now he returns to the charge and re-asserts the same repeatedly. I said before that I *had* given proof and reiterate it now most emphatically. Therefore, I cannot see how any further progrsss can be made in our controversy until this question of veracity is settled. And to settle it I send you the pamphlet he first criticised, and earnestly request you, in the interest of fair dealing, to publish the seven pages entire. The full force and value of that proof cannot be seen and appreciated without giving all the points that precede it, and the summing up of which it is.

I might, in conclusion, imitate the Doctor's noble and exalted poetical sentiments, and endeavor to settle such trifling questions as the true pathology and aetiology of Diphtheria, by paraphrasing his profound poem thus:

"Optics *dark* it takes, I ween
*Not* to see what's plain to be seen."

Or, I might return his ridicule, and leave the above named trifling questions to settle themselves, by saying he may be and evidently is, much more familiar with the truth in connection with "all dry-goods from dress-making hands," than he is with any scientific questions in connection with pathology. But I can't see that this helps those suffering and dying of Diphtheria, or the profession to a better understanding of this terrible disease, that they may treat it more successfully. My original pamphlet was as follows*:

There is such a diversity of opinion in the profession, upon the essential nature of Diphtheria, and its best method of treatment, that a few simple, but important facts require to be considered, in order that we may come to a clearer and better understanding, upon both the pathology and treatment of the disease.

In the first place, then, and in view of what is to follow, the fact should be borne distinctly in mind that the characteristic exudations of diphtheria, that is to say, all the membranes, or membranous formations of the disease, wherever they may organize, are wholly, or principally, fibrin. Of this fact there is no question. Oertel, Virchow and all other prominent writers upon the subject assert such to be the case; and we shall see as we proceed, that this fibrinous character of said membranes, gives us the key to one of the greatest mysteries of the disease. But before proceeding further with that part of our subject, we must consider in short, some of the leading characteristics of fibrin, as follows:

First. Fibrin in the proportion of two and two-tenths parts, in one thousand parts of blood, is one of the *natural* constituents of healthy blood.

Second. There never was a drop of healthy blood drawn, and

---

*From the Physicians' and Surgeons' Investigator of December, 1880.

left to itself a few minutes, that it did not coagulate and form a clot of the whole mass; and this clotting is *solely* due to the one part of fibrin in about five hundred parts of blood, fibrillating or organizing into minute threads or fibrils, which extend through all parts of the clot, in an interlacing network, that binds and holds the whole, not only of blood corpuscles, but even the water of the serum, together, for a time, in quite a firm mass.

Third. There is one way, and one way only, in which fibrin ever fibrillates to form a clot of blood, or organizes into a false membrane (the latter being considered further on), and that is, that particles of it first coagulate into very minute granules, and then these granules join themselves together and form exceedingly fine threads, which, in turn, interlace to form the clot, or a membrane, as the case may be.

With these few facts bearing upon the nature and action of healthy fibrin, before us, we can now go on and give our attention to a few equally simple and equally important facts in connection with diphtheria.

First, then, under this head, is the fact, that fibrin is always in excess in the blood in diphtheria, as it is in every other imflammatory disease—and generally in greater excess in diphtheria than in most other diseases characterized by the same degree of inflammation.

Second. When fibrin is in excess in the blood, it is *always* a source of more or less danger, as we all know to be the case in pleuritis, peritonitis, etc., and its excess is equally if not more dangerous in diphtheria than in most other diseases.

Third. One of the principal reasons why its excess is so dangerous in diphtheria, is that its natural tendency to coagulate is so great that when there is an accumulation in the circulation of three to four, or certainly of not more than four to five parts to one thousand parts of blood, there is imminent danger of its coagulating into large clots (thrombi) in the heart, that instantly take life, or into small clots, that are washed along by the current of blood until arrested by some smaller artery, when embolism is the result, and death by inflammation and a slower process almost as certainly follows. It is, indeed, in one of these ways that quite a goodly number of diphtheritic patients perish.

Fourth. When fibrin coagulates into heart-clots, or coagula of it of whatever size form in the circulation, it does so in only one way, and that the same as in the clotting of blood outside of the body, namely, by organizing first into minute granules, and these granules joining together into threads, interlacing through the mass, and making it more or less compact and firm in accordance with the degree of perfection of such fibrillation.

Fifth. Nature is conservative and preservative of life under all circumstances where she possibly can be. Hence, as fibrin is always in excess in the blood in diphtheria, and as all heart-clots and all false membranes of the disease are of fibrin, and the former, or heart-clots, are so extremely dangerous, there can be no question that the exudation and formation of all the membranes of the disease are the result of nature, or the preservative forces of life, stepping in and expelling the excess of fibrin from the blood to *prevent* its accumulation in the circulation to such an extent as to form coagula there that are so fatal.

Sixth. When the excess of fibrin is extravasated, or poured out of the blood-vessels upon the surface of the tonsils, or other parts, it rapidly organizes into the diphtheritic membrane; and in doing so, it here again exactly repeats the method of its clotting in healthy blood, or fibrillating into coagula in the circulation; namely, first into granules, and these into threads, and the latter into membranes.

Of course, it will be understood that, in the coagulation of healthy blood, the resulting mass will take the form of the receptacle that holds it; and also that the clots that form in the circulation are more or less in masses; whereas, when the fibrin is exuded in successive layers upon any surface, as is generally the case in diphtheria, it organizes into a membrane which will be of greater or less extent, and thicker or thinner, according to the quantity and extent of the exudation.

Seventh. Were the excess of fibrin not expelled from the circulation, *every* case of diphtheria would prove fatal in a few days from the formation of large coagula in the heart that would suddenly take life, or small ones that would lead to incurable inflammation, and cause death in that way. In pleuritis and peritonitis the excess of fibrin is generally poured out upon the surface of the pleura and peritoneum, to organize into false membranes there, and to avoid worse results in those diseases also, as in diphtheria. Therefore, let the membranes of diphtheria, although they are in themselves often so serious, be henceforth looked upon in their true light as the work of the conservative efforts of nature, to avoid thrombosis and embolism and a much more certainly fatal issue. And what is of equal importance let the treatment of this disease hereafter be more in accordance with these incontrovertible facts than it has been in the past, if we would avoid death instead of hastening it.

We can now enter upon a more intelligent discussion of that great mystery thrown around diphtheria by the assumed existence of the so-called bacteria, in connection therewith, than could be had without the foregoing facts before us. But we must first understand the characteristic and varying forms of these assumed bacteria. Oertel classifies them as follows:

1. "Sphærobacteria (spherical bacteria), *i. e.*, micrococcus.

2. "Microbacteria (rodlike bacteria), bacterium termo; less frequently, and only in the mouth and fauces, bacterium lineola.

3. "Spirobacteria (corkscrew-shaped bacteria), spirillum tenue, spirillum undula."

It should be understood, however, that there is not the slightest proof to show that these bodies are vegetable parasites or organisms, as assumed. The *forms* are there, it is true, and to be seen under the microscope, but every fact recorded of them, and taken in connection with what precedes, shows that they are simply and only the organizing stages of fibrin; or that the first of these three forms, namely, the spherical bacteria, or micrococci, are the molecular granules into which fibrin always first organizes in the healthy clot of blood, in the heart-clot, and in the membrane of diphtheria, as already described:

That the second form, or rod-like bacteria, are these same molecular granules, or others just like them, united together into threads or rod-like prolongations:

And that the third form, or spiral bacteria are the same, or similar threads of fibrin, which have contracted into more or less of the spiral shape under their firmer organization; and especially so if their ends have been left free from attachments that would hold them straight.

Therefore, there never was a drop of healthy blood coagulated that it did not develop these three forms of so-called bacteria, just the same as any case of diphtheria—the first two while the coagulum was forming, and for a short time after it was formed, and the last as soon as the clot commenced shrivelling by the contraction and curling up of the fibrils.

Such, then, is all there is of the great mystery of bacteria, which has exercised the profession so greatly for the past fifteen years, and which has led to treatment as false and fatal as the theory.

The limits of a journal article like this will not allow of one-twentieth the proof being given that there is to sustain the views here presented, but two or three points are offered to show that the facts must be as claimed.

That there is no evidence of the minute forms, developed in the blood and membranes of diphtheria, being vegetable parasites, as claimed, is shown by the following language of Oertel, vol. 1st, page 587, Ziemssen:

> "The vegetable organisms which have been observed in the diphtheritic membranes of the fauces and air passages, as well as in other products of the disease, belong to a group which comprises forms of such exceeding minuteness—for they stand upon the very borders of the visible—that, as yet, we possess only the most unsatisfactory knowledge of their nature and organization."

Now, after such a confession of "only the most unsatisfactory knowledge" upon the subject, let us apply to its solution the following facts given by Lehmann and Wood upon the action of fibrin under different circumstances, and see how plain and simple they render this whole question.

Lehmann, speaking of the coagulation of fibrin, Physiological Chemistry, vol. I, page 311, says:

> "If we trace this transition of the fibrin from the dissolved fluid condition into the solid state under the microscope, a careful observation shows us that the fresh *liquor sanguinis* exhibits nothing morphological beyond some few colorless blood corpuscles; when it begins to gelatinize, separate points or molecular granules appear at various spots, from which arise extremely fine straight threads, in radiating lines, although they do not form star-like masses, as in crystallization; these threads becoming elongated cross those springing from other solid points, until the whole field of view appears, as it were, covered with a delicate but somewhat irregular cobweb."

This furnishes the proof of the manner of coagulation of healthy fibrin, forming first minute granules and then threads, while all must know that the clots could not shrivel, as they always do, unless these threads contracted into more or less of the spiral form.

So much for the ordinary action of fibrin in forming a clot of blood: now let us consider a little of the proof of its demeanor in organizing false membranes: Wood, vol. I. page 28, says:

> "*Coagulable Lymph* is a name applied by English writers to a substance exuded from the vessels of an inflamed part, which, though it escapes in the liquid form, coagulates after exudation, and is capable of becoming organized, and thus forming a new living structure. This plastic substance appears, from chemical analysis, as well as from its physiological properties, to be closely analagous to, if it be not identical with the fibrin of the blood. It appears to be sometimes extravasated with little if any of the other constituents of the blood, but in general is mixed with serous fluid, and is probably thrown out of the blood vessels in the form of *liquor sanguinis* or fluid portion of the blood, deprived of the red corpuscles, and possibly somewhat altered in the process of exudation. Of this fluid, the fibrinous part concretes, while the albuminous portion remaining combined with water and saline matters in the form of serum, fills and distends the cavities and interstitial spaces into which it may have been effused, or escapes in the form of flux from exposed surfaces, or those having a natural outlet.

"All that is absolutely essential to the organization of the exuded fibrin is that it should be in contact with living tissue. As it first escapes it is a homogeneous, formless, transparent fluid; but very soon afterwards, if examined by the microscope, it is found to contain *multitudes of fibrils, great numbers of minute granules of different sizes*, and another set of minute bodies, which are often covered by a cellular envelope, and constitute what are called *exudation corpuscles.*"

The italics, excepting the first two and the last two words, are our own; and it might be explained in passing that the "exudation corpuscles" spoken of are simply decolorized blood-corpuscles.

Herein, then, we have the proof of the organization of plastic lymph, or fibrin, into false membranes on *any* living surface, upon which it is extravasated; which must include the membranes of diphtheria, they being always of fibrin. And in it we also have evidence of the fact claimed that the "homogeneous, formless, transparent fluid" that is first exuded, is "very soon afterwards, if examined by the microscope, found to contain multitudes of fibrils," and "great numbers of minute granules of different sizes," which are the forming stages of the fibrin into the membrane. Then if the fact is duly considered, that it is impossible to point out the slightest physical or other distinction between the *micrococci* of diphtheria and the molecular granules of fibrin in its false membranes, or between the so-called rod-like bacteria and the fibrils of said membranes, it would seem that our case is made out without further argument. If the fibrin is there, in forms precisely like the three forms of bacteria, as proven, all controversy upon the subject must be at an end.

Certainly no intelligent physician will, or can, reasonably claim that there are two sets of forms, exactly alike in size and appearance, in each stage of their development, alike as a whole, and alike in their consequences upon life; the one being vegetable and the other animal, and both present in almost infinite numbers in the exudations in every case of diphtheria. That would be absurd.

To my mind nothing in all the doings of men is more astonishing than that these facts have not been applied before. Probably there is not a prominent writer in all the numbers who have written upon diphtheria, who was not as familiar, and many of them more so, than the author, with the various facts given, as they stood isolated in medical literature; but the trouble has been the want of an *application* of them. And nothing shows more forcibly the terrible power of error over us all, when we get started wrong.

In conclusion, the cause of fibrin being brought into excess in the blood to produce the results shown, and the question of treatment, which is, after all, the more practical part of the subject, are far too complex for discussion here, and the best I can do now is to refer the reader to my late published work upon the subject. Of one thing, however, all may rest assured, that if we are to have any better results from treatment in the future, than in the past, the whole of it must be changed and made to conform to the true nature of the disease as it is, and not to any false conceptions we have had of it. But of all things let it hereafter be remembered that, if the excess of fibrin were not expelled from the blood vessels upon some surface, to be carried off in a fluid form, or to organize into a membrane, every case of diphtheria would be rendered fatal by said excess coagulating in the heart, or arteries; and that, in either case, the successful treatment must be that which will speedily reduce the excess of fibrin, and restore it to its normal proportion in the blood.

This is the paper that Dr. Wells first criticised, and I leave the reader to judge of the truth of his repeated assertions, that it contained no proof of what was claimed. As for his stale and shallow ridicule, I can afford to let that pass for just what it is, namely, very stale and very shallow; especially after showing, as I did before, that he was ignorant of the long-known fact, that fibrin is in excess in the blood in all inflammatory diseases; ignorant of the fact that the false membranes of diphtheria are composed of fibrin, and, finally, ignorant of the great danger that always exists in severe cases of diphtheria, of the fibrin coagulating in the heart, or arteries, and causing death through thrombosis or embolism. And here, for the present, I leave him.

